# CAMKs support development of acute myeloid leukemia

**DOI:** 10.1186/s13045-018-0574-8

**Published:** 2018-02-27

**Authors:** Xunlei Kang, Changhao Cui, Chen Wang, Guojin Wu, Heyu Chen, Zhigang Lu, Xiaoli Chen, Li Wang, Jie Huang, Huimin Geng, Meng Zhao, Zhengshan Chen, Markus Müschen, Huan-You Wang, Cheng Cheng Zhang

**Affiliations:** 1 Departments of Physiology and Developmental Biology, University of Texas Southwestern Medical Center, 5323 Harry Hines Boulevard, Dallas, TX 75390 USA; 2 Center for Precision Medicine, Department of Medicine, University of Missouri, 1 Hospital Drive, Columbia, MO 65212 USA; 3 Department of Hematology, The First Affiliated Hospital of Guangzhou Medical University, Guangzhou, China; 4 School of Life Science and Medicine, Dalian University of Technology, Liaoning, 124221 China; 5 Department of Electrical and Computer Engineering, Missouri University of Science and Technology, Rolla, MO 65409 USA; 6 Department of Laboratory Medicine, University of California San Francisco, San Francisco, CA 94143 USA; 7 Department of Systems Biology, Beckman Research Institute, Monrovia, CA 91016 USA; 8 Department of Pathology, University of California San Diego, La Jolla, CA 92093 USA

**Keywords:** Acute myeloid leukemia, CAMK, PirB, LILRB2, CREB, Leukemic stem cell

## Abstract

**Background:**

We recently identified the human leukocyte immunoglobulin-like receptor B2 (LILRB2) and its mouse ortholog-paired Ig-like receptor (PirB) as receptors for several angiopoietin-like proteins (Angptls). We also demonstrated that PirB is important for the development of acute myeloid leukemia (AML), but exactly how an inhibitory receptor such as PirB can support cancer development is intriguing.

**Results:**

Here, we showed that the activation of Ca (2+)/calmodulin-dependent protein kinases (CAMKs) is coupled with PirB signaling in AML cells. High expression of CAMKs is associated with a poor overall survival probability in patients with AML. Knockdown of CAMKI or CAMKIV decreased human acute leukemia development in vitro and in vivo. Mouse AML cells that are defective in PirB signaling had decreased activation of CAMKs, and the forced expression of CAMK partially rescued the PirB-defective phenotype in the MLL-AF9 AML mouse model. The inhibition of CAMK kinase activity or deletion of CAMKIV significantly slowed AML development and decreased the AML stem cell activity. We also found that CAMKIV acts through the phosphorylation of one of its well-known target (CREB) in AML cells.

**Conclusion:**

CAMKs are essential for the growth of human and mouse AML. The inhibition of CAMK signaling may become an effective strategy for treating leukemia.

**Electronic supplementary material:**

The online version of this article (10.1186/s13045-018-0574-8) contains supplementary material, which is available to authorized users.

## Background

Recently, we identified the human leukocyte immunoglobulin-like receptor B2 (LILRB2) and its mouse ortholog-paired Ig-like receptor (PirB) as receptors for several angiopoietin-like proteins (Angptls) [1]. LILRB2 and PirB are expressed by hematopoietic stem cells (HSCs) and leukemia stem cells in humans and mice, respectively, and support the ex vivo expansion of HSCs and acute myeloid leukemia (AML) development [1, 2]. The level of LILRB2 mRNA is significantly higher in human acute monoblastic and monocytic leukemia cells (M5 subtype of AML) than in other AML cells [1]. A deficiency of PirB in the MLL-AF9 and AML1-ETO9a AML mouse models resulted in increased differentiation and decreased self-renewal of leukemia stem cells and significantly downregulated expression of a large number of tumor-promoting genes [1]. Although it was known that PirB inhibits the differentiation of myeloid-derived suppressive cells to stimulate solid cancer development [3], our results indicate that certain immune inhibitory receptors can be expressed on cancer stem cells and play essential roles in inhibiting differentiation and supporting self-renewal of these cells.

LILRB2 contains four extracellular Ig-like domains and a cytoplasmic portion with three immunoreceptor tyrosine-based switch motifs (ITIMs) [4]. The mouse ortholog PirB has six extracellular Ig-like domains and four ITIMs in its intracellular domain. The LILRB2/PirB ITIMs recruit tyrosine phosphatases SHP-1 and SHP-2 [5–7]. We were intrigued as to how inhibitory receptors such as LILRB2 and PirB support stem cell activity and cancer development. We found that binding of Angptls to PirB/LILRB2 induces activation of tyrosine phosphatase SHP-1/SHP-2 and calcium/calmodulin-dependent protein kinase CAMKIV [1], both known to be critical for HSC repopulation and stimulation of leukemia development [1, 2, 8–10]. We also found that SHP can induce CAMK activation in AML cells [1, 11].

CAMKs are serine/threonine kinases that are activated by increases of intracellular calcium concentration and that mediate subsequent cellular activities. Few studies have shown that CAMKs play important roles in hematopoietic physiology and pathology. CAMKIV is required for HSC activity; *camk4*-knockout mice have defects in HSC survival and proliferation [10]. CAMKII suppresses differentiation and stimulates proliferation of myeloid leukemia cells [9]. CREB is one of the well-known targets of CAMKs in hematopoietic cells, which can be phosphorylated and activated by CAMKs. This signaling plays a critical role in both normal hematopoiesis and leukemogenesis [11–13].

In this study, we sought to investigate the function of CAMKs in AML cells and understand how CAMKs support PirB signaling in AML development. We found that the expression of several CAMKs inversely correlates with the overall survival of human AML patients. Knockdown of CAMKs decreased human myeloid leukemia development in vitro and in vivo. In the mouse MLL-AF9 primary AML cells, the defective PirB signaling decreased CAMK activation, and the forced expression of CAMKs partially rescued the PirB-defective phenotype. Importantly, the inhibition of CAMK kinase activity or deletion of CAMKIV significantly lowered the activity of AML stem cells. By rescue assay, we also identified the phosphorylation of CREB is critical downstream for CAMKIV signaling in AML cells. Our results indicate that the CAMK signaling supports self-renewal and inhibits apoptosis of AML cells.

## Methods

### Mice

C57 BL/6 and NOD*/*SCID*-*IL2RG (NSG) mice were purchased from the UT Southwestern Medical Center animal breeding core facility or Jackson Labs. The PirB TM mice [14] and CaMKIV-null mice [10] were purchased from MMRRC and Jackson Labs, respectively. Mice were maintained at the UT Southwestern Medical Center animal facility. All animal experiments were performed with the approval of UT Southwestern Committee on Animal Care and University of Missouri Committee on Animal Care.

### Antibodies, reagents, and PCR primers

Antibodies for flow cytometry, anti-Kit-APC, anti-Mac-1-APC, anti-Gr-1-PE, anti-CD3-APC, and anti-B220-PE, were purchased from BioLegend and used as described [1]. The manufacturers and catalog numbers for other antibodies and reagents are as follows: anti-PirB-PE, R&D Systems, FAB2754P; pCAMKI, Santa Cruz, sc-28438; anti-pCAMKII, Abcam, ab32678; anti-pCAMKIV, Santa Cruz Biotechnology, sc-28443-R; anti-CAMKI, Abcam, ab68234; anti-CAMKII, Cell Signaling, 4436; anti-CAMKIV, Cell Signaling, 4032; anti-pCREB, Cell Signaling, 9198S; anti-CREB, Cell Signaling, 9197S; anti-actin, Sigma Aldrich, A2066; STO-609, Sigma Aldrich, S1318; KN93, Sigma Aldrich, K1385; The PCR primer sequences were as follows: hCAMKI forward: CGGAGGACA TTAGAGACA, reverse: CTCGTCATAGAAGGGAGG-3; hCAMKIV forward: GATGAAAGAGGCGATCAG, reverse: TAGGCCCTCCTCTAGTTC. PirB forward: GAG AATCACCAGACACATGC, PirB reverse: CTGCCCTCATGTCTTAACTT, mCAMKIV forward: AAGCAGGCGGAAGACATTAGG, CAMKIV reverse: AGTTTCTGAGTCCTCTTGTCCT.

### Virus infection and leukemia cell transplantation

For retrovirus production, human embryonic kidney 293T cells were grown in DMEM with 10% fetal bovine serum (FBS) and transfected by MSCV-MLL-AF9-IRES-YFP, MSCV-CAMKIV-IRES-GFP, MSCV-CAMKI-IRES-GFP, or MSCV-CREB-IRES-GFP encoding plasmids and pCL-ECO to produce retroviruses. The infection of Linage-negative cells with retrovirus was performed as described previously [1, 15, 16]. Briefly, we infected Lin^−^ cells with retroviral supernatant in the presence of 8 μg/mL polybrene. After infection, these cells were incubated overnight in medium with 10% FBS, 20 ng/mL SCF, 10 ng/mL IL-3, and 10 ng/mL IL-6. Infected cells (300,000) were transplanted into lethally irradiated (1000 rad) WT mice by retro-orbital injection. For secondary transplantation, YFP^+^ bone marrow (BM) cells from primary transplanted mice were sorted and transplanted into mice with 200,000 normal BM cells as competitors.

For lentivirus production, the lentiviral vector Pll3.7 was used to express shRNAs to target CAMKI (sense: TGCCAGAGAATCTGCTGTACTATTCAAGAGATAGTA CAGCAGATTCTCTGGCTTTTTTC; antisense: GAAAAAAGCCAGAGAATC TGCTGTACTATCTCTTGAATAGTACAGCAGATTCTCTGGCA) and CAMKIV (sense: TGATATTACAGTGAGCGAGATGTTCAAGAGACATCTCGCTCACTGTAATATCTTTTTTGGAAC; antisense: TCGAGTTCCAAAAAAGATATTACAGTGAGCGAGATGTCTCTTGAACATCTCGCTCACTGTAATATCA). The lentivirus plasmid, pSPAX2, and pMD2.G (4:3:1) were transfected using PolyJet™ In Vitro DNA Transfection Reagent (SignaGen Laboratories) into 293T cells. The resulting virus supernatant was collected 48–72 h later.

For Crisper-Cas9 system, we used pCW-Cas9 (gift from Eric Lander & David Sabatini (Addgene plasmid # 50661) [17]) to make stable MV4-11-Cas9 cell line, and designed three gRNA targeting the CAMK1 (gRNA1-GCTACGACTTCCGAGATGTTC; gRNA2-GGAGCTCTTTGACCGTATTGG; gRNA3-GATCCCGGTGTACAATGCCC) and one scramble gRNA (GAACGACTAGTTAGGCGTGTA).

### Flow cytometry, immunohistochemistry, and cytospin assays

We performed flow cytometry, immunohistochemistry, and cytospin as described previously [1, 15, 16]. For flow cytometry analysis of AML cells, peripheral blood or BM cells were stained with anti-Mac-1-APC, anti-Gr-1-PE, anti-CD3-APC, anti-B220-PE, or anti-Kit-PE monoclonal antibodies (BioLegend). For analysis of apoptosis, indicated AML cells were stained with PE-conjugated anti-annexin V and 7-AAD (BD Pharmingen) according to the manufacturer’s instructions.

### Human AML mouse xenograft model

Human leukemia cell lines were cultured in RPMI supplemented with 10% FBS at 37 °C in 5% CO_2_ and the normal level of O_2_. Human leukemia cell lines were from ATCC. Xenografts were performed essentially as we described [11, 18]. Briefly, adult NSG mice (6–8 weeks old) were sub-lethally irradiated with 250 cGy total body irradiation prior to transplantation. ShRNA-infected cells were resuspended in 200 μl PBS containing 1% FBS at a final dose of 1 × 10^6^ human GFP^+^ viable cells per mouse for retro-orbital injection. One to 4 months after transplantation, the peripheral blood (PB), BM, spleen, and liver were assessed for engraftment by flow cytometry.

### CFU assays

Five hundred YFP^+^Mac-1^+^Kit^+^ BM cells from AML mice were plated in colony-forming unit (CFU) medium (M3534, Stem Cell Technologies) for CFU-GM assays, according to the manufacturer’s protocols [1, 18]. After 7 days, 2000 cells from three dishes were isolated for secondary plating.

### Patient overall survival analysis

Data were obtained from the TCGA AML database (https://tcga-data.nci.nih.gov/docs/publications/tcga/; accessed September 5, 2013), and levels were normalized to *GADPH* expression. Patients were divided into two groups based on whether their expression was above or below the median level of a gene.

### Western blotting

Cell lysates (100 μg samples) were separated by electrophoresis on a 4–12% SDS-polyacrylamide gel (BioRad), and the proteins were transferred onto a nitrocellulose membrane. The membrane was probed with indicated primary antibody for 1 hour at room temperature and then incubated with horseradish peroxidase-conjugated secondary antibody, which was detected with the chemiluminescence SuperSignal kit (Pierce).

### Statistical analyses

Data are expressed as mean ± SEM. For all experiments except determination of survival, data were analyzed by Student’s *t* test and differences were considered statistically significant if *p* < 0.05. The survival rates of the two groups were analyzed using a log-rank test, and differences were considered statistically significant if *p* < 0.05.

## Results

### CAMK activities are regulated by PirB signaling in AML development

**Fig. 1:**
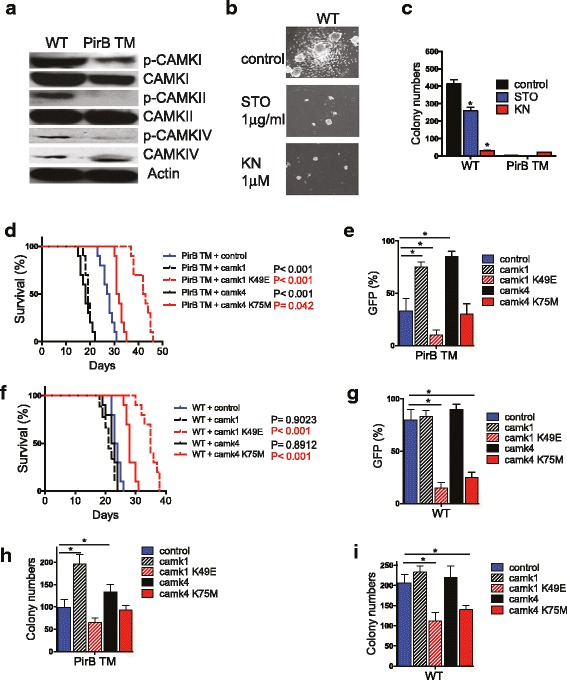
Camk transduction enhances PirBTM MLL-AF9 AML development. **a** Phosphorylation of CAMKI, CAMKII, and CAMKIV was decreased in the PirBTM MLL-AF9 AML BM cells compared to WT cells. **b**, **c** Colonies formed from WT or PirB TM AML cells upon CAMK or CAMKK inhibitor treatment. Numbers of colonies formed by WT AML cells are decreased by addition of STO609 (STO) or KN93 (KN) (*n* = 3). **d**, **e**, **h** Retrovirally expressed *Camk1* and *Camk4*, but not *Camk1* mutant (K49E) or *Camk4* mutant (K75M), rescued PIRB TM phenotype upon secondary transplantation. Retrovirally expressed *Camk1* and *Camk4* had similar levels as endogenous proteins in WT controls (Additional file 1: Figure S1). **d** Survival curves of mice transplanted with 3000 of these ectopically *Camk1*-expressing, *Camk4-*expressing, *Camk1* K49E-expressing, *Camk4* K75M-expressing, or control cells (*n* = 15 mice). **e** Percentages of retrovirus-infected (GFP^+^) AML cells in PB of secondary recipient mice after 28 days of transplantation. (*n* = 5 mice). **h** CFU numbers of retrovirus-infected (GFP^+^) AML cells in colony-forming assays. The experiment was repeated three times with similar results. **f**, **g**, **i** Retrovirally expressed *Camk1* and *Camk4* cannot change WT AML phenotype upon second transplantation. **f** Survival curves of mice transplanted with 3000 WT AML cells of these ectopically *Camk1*-expressing, *Camk4*-expressing, *Camk1* K49E-expressing, *Camk4* K75M-expressing, or control cells (*n* = 15 mice). **g** Percentages of retrovirus-infected (GFP^+^) AML cells in PB of secondary recipient mice after 28 days of transplantation. (*n* = 5 mice). **i** CFU numbers of retrovirus-infected (GFP^+^) AML cells in colony-forming assays. The experiment was repeated three times with similar results; *p < 0.05

Because LILRBs are highly expressed by monocytic AML (M5) cells [1], we used a retroviral MLL-AF9 transplantation mouse M5 AML model [19, 20] to investigate the relationship between PirB signaling and CAMKs in AML development. PirBTM mice [14], in which four exons encoding the transmembrane domain and part of the intracellular domain of *PirB* are deleted, were used to provide PirB-defective cells. PirBTM and control wild-type (WT) cells infected with MSCV-MLL-AF9-IRES-YFP retrovirus were transplanted to establish AML mice. We sought to determine whether the CAMK family decreased expression/activities in the PirB-defective MLL-AF9 AML mouse model. Compared to WT controls, PirBTM cells from MLL-AF9 AML mice had significantly decreased phosphorylation of CAMKI, CAMKII, and CAMKIV (Sun et al. [22]) (Fig. 1a), suggesting that CAMK activities are regulated by the PirB signaling pathway.

To test whether CAMK activities are essential to AML cells, we treated primary mouse MLL-AF9 AML cells with the CAMKK inhibitor STO-609 and CAMK inhibitor KN93. Both inhibitors diminished colonies in WT AML cells instead of PirB-deficient AML cells (Fig. 1b, c). These data show that the kinase activity of CAMK regulated by PirB signaling is critical for the growth of AML cells.

### CAMK transduction rescues PirB defects in AML

We used gain-of-function approaches to further determine the role of CAMKI and CAMKIV in PirB signaling in AML cells. We introduced retroviruses encoding CAMKI or CAMKIV into PirB-defective AML cells to study whether CAMKs could rescue PirB defects in AML development. Mice transplanted with MLL-AF9-transduced PirBTM cells developed AML more slowly and survived longer than those transplanted with PirBTM cells that overexpressed CAMKI or CAMKIV (Fig. 1d). The PirBTM leukemia cells that overexpress CAMK exhibited accelerated development as demonstrated by the twofold increase in leukemia cell infiltration in the peripheral blood. This was detected using flow cytometry of these mice, compared to mice transplanted with PirBTM cells that did not overexpress CAMK, at 28 days post-transplantation (Fig. 1e). The in vitro CFU assay also showed both CAMKI and CAMKIV can increase PirBTM AML cells’ colony-forming ability (Fig. 1h). Interestingly, in all of the rescue assays (Fig. 1d, e, h), neither the kinase-inactive CAMKI mutant (K49E) nor the CAMKIV mutant (K75M) was capable of supporting PirBTM leukemia development. We also performed the rescue assays in WT leukemia cells. Though CAMK overexpression did not affect WT leukemia development, the kinase-inactive CAMK1 mutant and CAMKIV mutant were able to play dormant negative roles in leukemia development in vivo and in vitro (Fig. 1f, g, i)*.* These results demonstrate that CAMK, depending on their kinase activity, can rescue PirB defects in AML development, supporting our hypothesis that CAMKs are downstream mediators of PirB signaling.

### CAMKIV supports mouse AML development during serial transplantation

To gain a deeper understanding of the mechanism by which CAMKs support AML development, we sought to examine AML development in genetic CAMK deletion model. While CAMKI and CAMKII have multiple isoforms, CAMKIV exists as a single form. The availability of the *Camk4*-null mice [10] allowed us to focus on the role of CAMKIV as a representative of the CAMK family in the mouse AML model. It is clear that PirB and CAMKIV are highly expressed by hematopoietic progenitors and MLL-AF9 AML stem cell-enriched Mac-1^+^Kit^+^ population [11, 20, 21] (Additional file 1: Figure S2a-c).

**Fig. 2:**
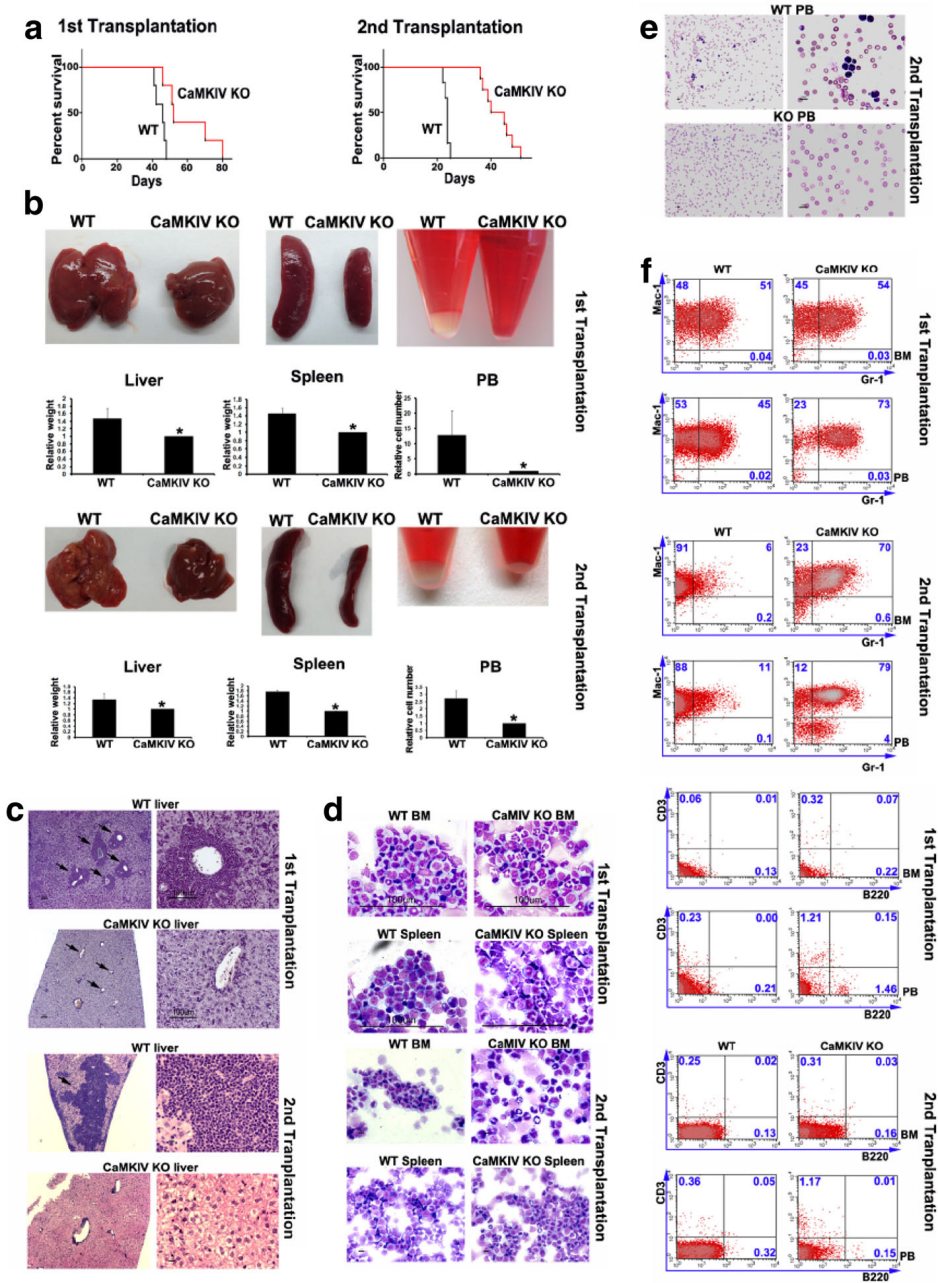
Loss of CAMKIV decreases MLL-AF9 AML development. **a** Mice transplanted with MLL-AF9-infected WT hematopoietic progenitors had significantly reduced survival compared to mice transplanted with MLL-AF9-infected *Camk4*-null hematopoietic progenitors in both primary (*p* = 0.0019) and secondary (*p* = 0.0021) transplantation (*n* = 16). **b** Comparison of the sizes of spleens and livers and numbers of peripheral blood cells of the mice transplanted with WT MLL-AF9 cells and those with *camk4*-null MLL-AF9 cells at 41 days after first transplantation and 23 days after second transplantation. **c** Histological analysis of AML infiltration in the livers of mice transplanted with control or CAMKIV-transduced PirBTM AML cells (hematoxylin/eosin staining). Significant differences in AML infiltration to the liver in samples are indicated by arrows. **d**, **e** Representative Wright-Giemsa-stained cytospin preparation of the BM, spleen, and peripheral blood cells from leukemic mice. **f** Representative flow cytometry plots show that *camk4*-null AML mice have more differentiated cells in BM and peripheral blood (PB) compared to mice transplanted with WT cells, at 23 days after transplantation. *p < 0.05, scale bar is 100 μM

The mice transplanted with MLL-AF9-transduced *Camk4*-null cells developed AML significantly more slowly than controls in both primary and secondary transplantation (Fig. 2a). The delayed development of the *Camk4*-null AML was also evident from the significantly decreased liver and spleen sizes and the numbers of white blood cells in circulation in the mice transplanted with *Camk4*-null cells compared to controls (Fig. 2b). The infiltration of *Camk4*-null myeloid leukemia cells into the liver and spleen was significantly decreased (Fig. 2c). There were more mature and differentiated hematopoietic cells especially the Gr-1^+^ myeloid cells in mice that received *Camk4*-null cells than in those given control WT cells, based on cytospin and flow cytometry analyses (Fig. 2d–f). These results demonstrated that a lack of CAMKIV results in slower AML development.

**Fig. 3:**
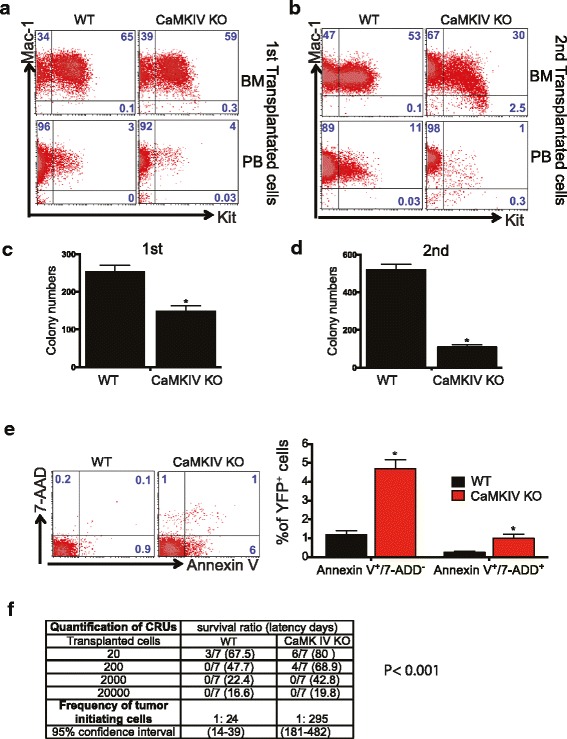
Loss of CAMKIV decreases self-renewal capacity of AML-SCs. **a**, **b** Representative flow cytometry plots show that *Camk4*-null MLL-AF9 AML mice had decreased numbers of YFP^+^Mac-1^+^Kit^+^ progenitors in both primary and secondary transplants. **c**, **d** Primary and secondary transplants with *Camk4*-null MLL-AF9 BM cells show significantly decreased colony-forming ability (*n* = 3). **e** *Camk4*-null MLL-AF9 BM cells show increased apoptosis. AnnexinV^+^/7AAD^−^ indicates early apoptosis, and AnnexinV^+^/7AAD^+^ indicates late apoptosis. *p < 0.05. **f** Limiting dilution assays comparing the frequencies of AML stem cells in WT and *Camk4*-null MLL-AF9 AML. The indicated YFP^+^ WT and *Camk4*-null MLL-AF9 BM cells that were collected from primary recipients were co-transplanted with 2 × 10^5^ bone marrow competitor cells into lethally irradiated recipients. The CRUs were calculated by L-Calc software

We further analyzed whether CAMKIV affects AML stem cell (AML-SC) activity. Primary transplants with *Camk4*-null cells did not have significant differences in YFP^+^Mac1^+^Kit^+^ cells from mice transplanted with WT cells (Fig. 3a). In drastic contrast, the YFP^+^Mac1^+^Kit^+^ cells in secondarily transplanted mice decreased from 53 to 30% in BM and from 11 to 1% in peripheral blood (Fig. 3b). A serial plating CFU assay was performed to test the self-renewal of AML-SCs in vitro. The deficiency of CAMKIV decreased CFUs in both primary plating and secondary plating, with dramatically lower numbers of colonies in secondary plating (Fig. 3c, d). Accordingly, CAMKIV was capable of rescuing the defects of PirBTM AML cells (Fig. 1h).

We also measured the apoptosis of *Camk4*-null and control AML BM cells. Overall, the MLL-AF9 BM AML cells had very low levels of early and late apoptosis (1.2 and 0.1%, Fig. 3e). Levels of apoptosis were dramatically increased in the *Camk4*-null AML BM cells (4.6 and 0.9%, Fig. 3e). These results suggest that CAMKIV supports the survival of AML cells. This is consistent with the report that CAMKIV is a critical survival factor for HSCs [10].

To quantitate how CAMKIV deficiency affects the AML-SC frequency, we performed transplantations by limiting dilution of sorted YFP^+^ WT and *Camk4*-null MLL-AF9 BM cells, collected from primary recipients. Leukemia development, as measured by survival ratio and latency days, is summarized in Fig. 3f. The frequency of AML-SCs was assessed and plotted as the percentages of non-leukemia-developing mice. Strikingly, the frequency of functional AML-SCs in *Camk4*-null primary MLL-AF9 AML model mice was only 1/12 (= 24/295) of that in control WT AML mice. This limiting dilution transplantation assay confirms that CAMKIV supports the activity of AML-SCs.

### CAMKs are essential for the growth of human leukemia cells

**Fig. 4:**
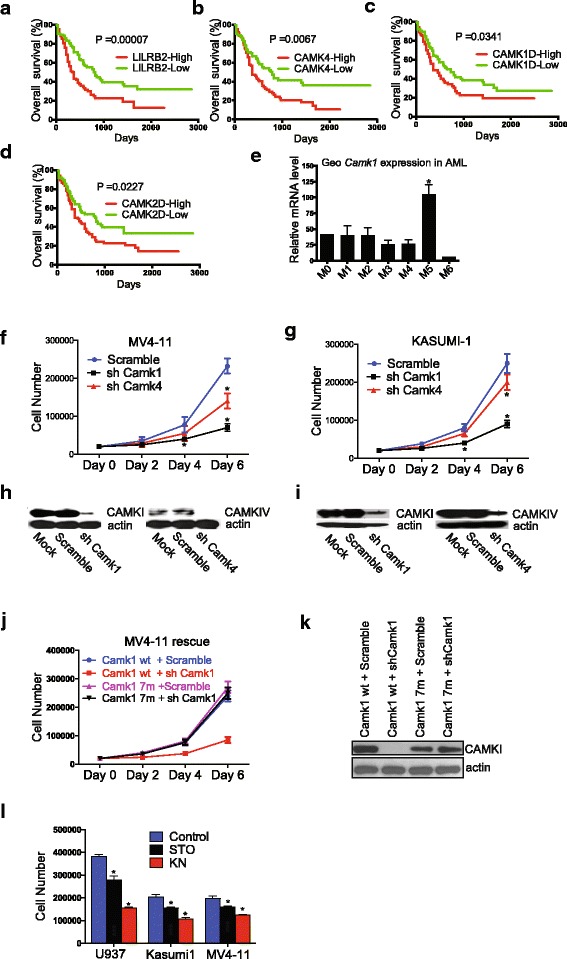
CAMKs play critical roles in human AML. **a–d** LILRB2 and CAMK expression negatively correlates with the overall survival of AML patients. The mRNA expression data of LILRB2 and CAMKs were obtained from the TCGA AML database (https://tcga-data.nci.nih.gov/docs/publications/tcga/; accessed November 5, 2012) and normalized by GADPH expression. Patients were separated into two groups based on whether they have higher (*n* = 83) or lower (*n* = 82) than the average expression levels of the indicated genes (*n* = 186). **e** An in silico analysis of human *Camk1* mRNA expression in 43 human AML samples as described previously [24]. **f** Treatment with shRNAs targeting *Camk1 or Camk4* inhibited the growth of MV4-11 cells. GFP^+^ cells were sorted by flow cytometry 1 day post-infection, and 20,000 cells were plated. Cell numbers were determined at three time points (days 2, 4, and 6) from triplicate wells. The experiment was repeated three times with similar results. **g** Inhibition of *Camk1* or *Camk4* expression inhibited the growth of KASUMI-1 cells. Cell numbers were determined at three time points (days 2, 4, and 6) from triplicate wells. The experiment was repeated three times with similar results. Knockdown of Camk1 and Camk4 in MV4-11 cells and KASUMI-1 cells as determined by Western blotting (**h**, **i**). **k** Rescue of *Camk1*-knockdown phenotype. Seven mutations were introduced into puromycin-seleted *Camk1* expression lentivirus vector. Expression from this mRNA did not change CAMK1 amino acid sequence and was not silenced by shRNA *Camk1*. Mutant *Camk1* (7 m) infected MV4-11 cells were resistant to the shRNA-*Camk1*-induced growth inhibition. The experiment was repeated three times with similar results. GFP^+^ cells were sorted by flow cytometry 1 day post-infection, and 20,000 cells were plated. Cell numbers were determined on days 2, 4, and 6 in triplicate wells. The experiment was repeated three times with similar results. **l** Western blot showed mutant *Camk1* could not be silenced by shRNA *Camk1*. **m** Cell growth of human AML cells (U937, Kasumi1 and MV4-11) were inhibited upon CAMK (STO609) or CAMKK inhibitor (KN93) treatment. Numbers of cells were calculated 6 days post treatment; *p < 0.05

Previously, we showed that the activation of LILRB2 induced CAMK activation. Similarly, based on our in silico analysis of human samples, the expression of LILRB2 and several CAMKs correlate inversely with the overall survival of AML patients (Fig. 4a–d), and CAMK1 is highly expressed by M5 AML cells (Fig. 4e) as LILRB2 [1]. These results suggest that CAMKs may play important roles in AML development. The activation of CAMKIV in human myeloid cells including the monoblastic KASUMI-1 cells and MLL-rearranged MV4-11 cells is greater than that in lymphoblastic leukemia cells such as KASUMI-2 and SUP-B15 cells (Additional file 1: Figure S3). The knockdown of CAMK1 or CAMK4 in AML cell lines including MV4-11 cells and KASUMI-1 led to significant inhibition of cell growth (Fig. 2g–j), indicating that CAMKs are functionally essential for human leukemia cells. By performing rescue assays (Fig. 4k), and Crisper assays (Additional file1: Figure S4) in MV4-11 cells, we proved the specific effect of CAMKI deficiency on AML cell growth.

**Fig. 5:**
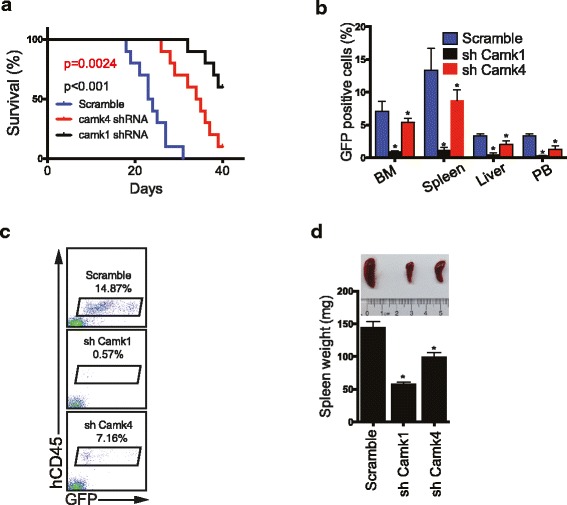
Knockdown of CAMK1 or CAMK4 blocks xenograft of human leukemia cells. **a** Survival curve of NSG mice transplanted with MV4-11 cells (1 × 10^6^ cells) infected with virus designed to express GFP and either scrambled shRNA, *Camk1* shRNA, or *Camk4* shRNA. GFP^+^ cells were collected and transplanted into *NSG* mice 1 day post-infection (*n* = 10 mice). **b** Percentages of GFP^+^ cells in BM, spleen, liver, and PB at 28 days after transplantation. **c** Representative flow cytometry plots indicating decreased spleen engraftment of MV4-11 cells treated with shRNA targeting *Camk1* or *Camk4*. Staining with anti-human CD45 antibodies confirmed engraftment was from transplanted human leukemia cells. The percentages of hCD45^+^ population are indicated. **d** Comparison of the sizes of spleens of the mice transplanted with control MV4-11 leukemia cells or cells expressing shRNA targeting *Camk1* or *Camk4* (*n* = 5 mice); *p < 0.05

Next, we tested the in vivo effect of knockdown of CAMK1 *or* CAMK4 expression in MV4-11 leukemia cells in transplanted *NOD/SCID-IL2RG (NSG)* mice. Both *Camk1* or *Camk4* knockdown significantly prolonged the survival of xenografted mice (Fig. 5a) and greatly inhibited leukemia development as determined by analysis of knockdown cells (Fig. 5b), human leukemic hCD45^+^ cells (Fig. 5c), and spleen size (Fig. 5d).

### CREB mediates the effect of CAMKIV on AML

**Fig. 6:**
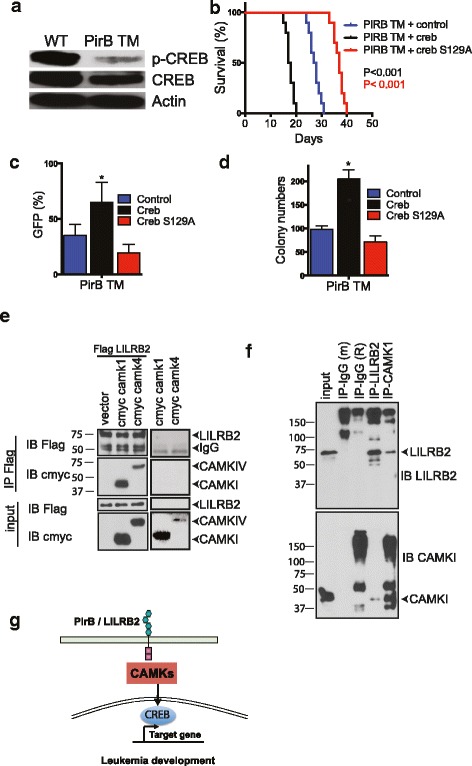
CREB activity plays important role in PirB/LILRB2-CAMKs signaling in AML-SCs. **a** Phosphorylation of CREB was decreased in the PirBTM MLL-AF9 AML BM cells compared to control cells. **b**–**d** Retrovirally expressed *Creb*, but not *Creb* mutant (S129A), rescued PirBTM phenotype upon secondary transplantation. Retrovirally expressed *Creb* had similar levels as endogenous proteins in WT controls. **b** Survival curves of mice transplanted with 3000 of these ectopically *Creb*-expressing, *Creb* S129A-expressing, or control cells (*n* = 10 mice). **c** Percentages of retrovirus-infected (GFP^+^) AML cells in PB of secondary recipient mice after 28 days of transplantation. (*n* = 5 mice). **d** CFU numbers of retrovirus-infected (GFP^+^) AML cells in colony-forming assays. The experiment was repeated three times with similar results; **e** CAMKI and LILRB2 bound in transfected 293T cells. The indicated flag-tagged LILRB2 or myc-tagged CAMKI or CAMKIV proteins were overexpressed in 293 cells. Flag antibody was used to precipitate LILRB2 proteins, and the flag or myc antibodies were used in Western blots. **f** Endogenous CAMKI and LILRB2 interact with each other as determined by bidirectional pull-down assays. MV4-11 cells (1 × 10^7^ cells) were lysed with transmembrane protein extraction reagent and indicated antibodies were used for immunoprecipitation and Western blot; *p < 0.05. **g** Schematic summary of the signaling pathway mediated by the PirB/LILRB2-CAMKs-CREB in AML cells

CREB was previously reported to be a possible downstream target of CAMKIV [22]. We found that PirBTM AML cells have decreased phosphorylation of both CAMKIV and CREB (Fig. 1a, Fig. 6a). Therefore, we hypothesized that CREB is a downstream mediator of PirB signaling in AML cells. To test this hypothesis, we introduced retrovirally expressed WT or inactive mutant CREB (S129A) into PirBTM AML cells. WT but not S129A CREB could rescue the defective phenotype of PirB TM AML cells in vitro and in vivo (Fig. 6b–d). These results suggest that CREB acts downstream of PirB-mediated signaling to support AML development.

### LILRB2 and CAMKs interact with each other in AML cells

To test the relationship between LILRB2 and CAMKs, we transfected Flag-tagged LILRB2 and Myc-tagged CAMKI or CAMKIV into 293T cells, then performed co-immunoprecipitation and Western blotting assays. Both CAMKI and CAMKIV bind LILRB2 in transfected 293T cells (Fig. 6e). Importantly, our bidirectional pull-down experiments showed that endogenous LILRB2 and CAMK1 interact with each other in MV4-11 cells (Fig. 6f). These data further support the conclusion that CAMKs play a crucial role in LILRB2-mediated signaling in AML cells (Fig. 6g).

## Discussion

Recently, we showed that Angptl binding to human LILRB2, or the mouse homolog PirB, stimulates phosphorylation of SHP-2 and CAMKIV [1, 2]. We sought to understand how immune inhibitory receptors support stem cell activity and cancer development. Here, we demonstrated that CAMKs play critical roles in sustaining AML-SC activity and PirB supports AML development at least partially through activation of CAMKs. To our knowledge, this is the first demonstration of how an ITIM-containing receptor signals, via activation of kinase activities, to support cancer development.

The positive roles of CAMKs in mouse and human leukemia development are supported by a variety of evidence as shown in our study. (1) The expression of CAMKs inversely correlates with the overall survival of AML patients. (2) Knockdown of CAMKI or CAMKIV inhibits growth of human AML cells in vitro and in vivo. (3) CAMKI and CAMKIV rescue PirB deficiency in a mouse AML model, and deletion of CAMKIV decreased the activity of AML-SCs. (4) CAMKIV supports the development of acute lymphoblastic leukemia in a mouse model (unpublished data). These results are in agreement with the previous reports that CAMKIV is required for HSC activity and CAMKII suppresses differentiation and stimulates proliferation of myeloid leukemia cells [10]. Because we used the MLL-AF9 fusion oncogene to initiate AML in the mouse model, our results suggest that CAMKs play a critical role in the maintenance and progression of AML.

Because CAMKs network with many different signaling pathways, and the signaling of ITAM or ITIM receptors is rather divergent [23], it is possible that CAMKs only represent a critical branch of the total signaling cascades linked to LILRB2/PirB. In general SHP-1 and SHP-2 act immediately downstream of an ITIM-containing inhibitory receptor such as LILRB2/PirB to initiate further signaling to regulate cell fates. While we showed that SHP-1/2 can induce CAMK activation in AML cells [1, 11], it will be important to determine whether other signaling molecules act downstream of LILRB/SHP in leukemia cells. In addition, although we can use serial transplantation to measure AML-SC function and self-renewal, because AML-SCs were not purified to homogeneity in our study, it remains to be determined whether this signaling network differs in stem cells and in more differentiated AML cells.

## Conclusion

We demonstrated that CAMKs are important signaling molecules to support self-renewal and survival in AML cells. Our results thus revealed one mechanism by which the ITIM-containing inhibitory receptors support stem cell activity and AML development and also have important implications in development of new therapeutic strategies in leukemia treatment. It is known that deletion of PirB from normal hematopoiesis or adult HSCs does not have significant in vivo effects [1], and *Camk4*-knockout mice had only minor defects in HSCs [10]. By contrast, the loss of function of LILRB2/PirB or CAMKs are detrimental to AML development. It is therefore likely that inhibition of CAMKs will be effective in treating leukemia with minimal side effects in the hematopoietic system.

## Additional file

Additional file 1: **Figure S1.** Western blot showed expression level of CAMKI and CAMKIV in each samples of Fig. 1d. **Figure S2.** CaMKIV is highly expressed in AML-SC enriched population. (a-b) The expression of PirB (a) and CaMKIV (b) in different normal BM populations as determined by real-time RT-PCR (*n* = 3). (c) CaMKIV expression in total, YFP^+^Mac-1^+^Kit^−^, and YFP^+^Mac-1^+^Kit^+^ BM AML cells as determined by real-time RT-PCR (n = 3). *p < 0.05. Error bars, s.e.m. **Figure S3.** Phosphorylation of CAMKIV is greater in myeloid cell lines (U937 and MV4-11) than in ALL cell lines (KASUMI-2 and SUP-B15). **Figure S4.** MV4-11 cell growth was inhibited by three different gRNAs targeting camk1. Cell numbers were calculated four days post doxycycline (DOX 1μg/ml) inducement (a). Western blot showed silent effect of gRNAs targeting CAMK1 (b). (PDF 660 kb)

## Abbreviations

AML: Acute myeloid leukemia
Angptls: Angiopoietin-like proteins
CAMK: Ca(2+)/calmodulin-dependent protein kinases
ITIM: Immunoreceptor tyrosine-based switch motif
LILRB2: Leukocyte immunoglobulin-like receptor B2
PirB: Ortholog-paired Ig-like receptor
WT: Wild-type
