# Supplementary figures and images for: CAMKs support development of acute myeloid leukemia
Source: J Hematol Oncol. 2018 Feb 27;11:30. doi: 10.1186/s13045-018-0574-8 (PMC5828341; doi:10.1186/s13045-018-0574-8)

Supplementary Figure 1

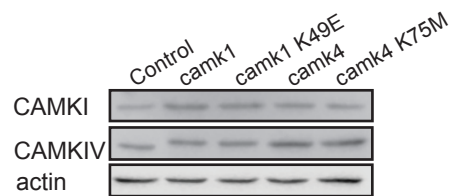

Supplementary Figure 2

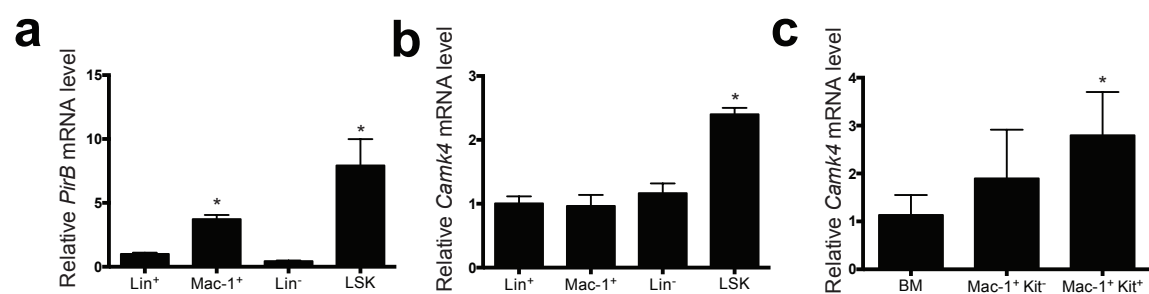

Supplementary Figure 3

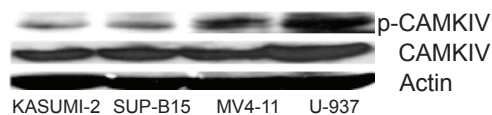

Supplementary Figure 4

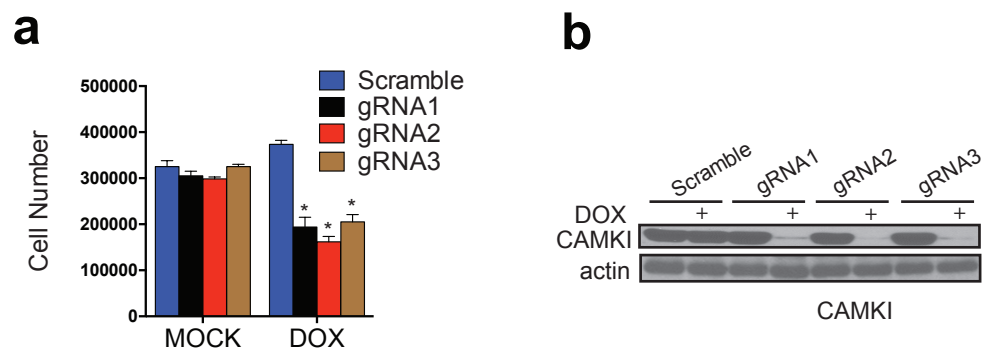

Supplement: Supplementary file 1 — Figure S1. Western blot showed expression level of CAMKI and CAMKIV in each samples of Fig. 1d. Figure S2. CaMKIV is highly expressed in AML-SC enriched population. (a-b) The expression of PirB (a) and CaMKIV (b) in different normal BM populations as determined by real-time RT-PCR (n = 3). (c) CaMKIV expression in total, YFP+Mac-1+Kit−, and YFP+Mac-1+Kit+ BM AML cells as determined by real-time RT-PCR (n = 3). *p < 0.05. Error bars, s.e.m. Figure S3. Phosphorylation of CAMKIV is greater in myeloid cell lines (U937 and MV4-11) than in ALL cell lines (KASUMI-2 and SUP-B15). Figure S4. MV4-11 cell growth was inhibited by three different gRNAs targeting camk1. Cell numbers were calculated four days post doxycycline (DOX 1μg/ml) inducement (a). Western blot showed silent effect of gRNAs targeting CAMK1 (b). (PDF 660 kb) [file 13045_2018_574_MOESM1_ESM.pdf]
